# 2-Amino­pyridinium 4-methyl­benzoate dihydrate

**DOI:** 10.1107/S160053680803153X

**Published:** 2008-10-04

**Authors:** Yun Liu, Jie Li

**Affiliations:** a Basic Experiment Teaching Center, Henan University, Kaifeng 475004, People’s Republic of China

## Abstract

The crystal structure of the title salt, C_5_H_7_N_2_^+^·C_8_H_7_O_2_^−^·2H_2_O, contains a three-dimensional supra­molecular framework constructed through N—H⋯O and O—H⋯O hydrogen bonds.

## Related literature

For a related structure, see: Wang & Wei (2005 ▶).
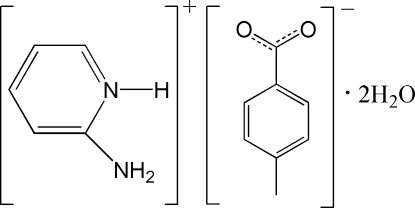


## Experimental

### 

#### Crystal data

C_5_H_7_N_2_^+^·C_8_H_7_O_2_^−^·2H_2_O
*M* *_r_* = 266.29
Monoclinic, 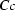

*a* = 12.2059 (14) Å
*b* = 13.1531 (16) Å
*c* = 8.9937 (11) Å
β = 96.617 (2)°
*V* = 1434.3 (3) Å^3^
*Z* = 4
Mo *K*α radiation
μ = 0.09 mm^−1^
*T* = 296 (2) K
0.23 × 0.18 × 0.16 mm

#### Data collection

Bruker SMART CCD diffractometer
Absorption correction: multi-scan (*SADABS*; Sheldrick, 2001 ▶) *T*_min_ = 0.979, *T*_max_ = 0.985
4203 measured reflections
1567 independent reflections
1406 reflections with *I* > 2σ(*I*)
*R*_int_ = 0.019

#### Refinement

*R*[*F* ^2^ > 2σ(*F* ^2^)] = 0.032
*wR*(*F* ^2^) = 0.089
*S* = 1.04
1567 reflections
175 parameters
8 restraints
H-atom parameters constrained
Δρ_max_ = 0.12 e Å^−3^
Δρ_min_ = −0.12 e Å^−3^

### 

Data collection: *SMART* (Bruker, 2001 ▶); cell refinement: *SAINT-Plus* (Bruker, 2001 ▶); data reduction: *SAINT-Plus*; program(s) used to solve structure: *SHELXS97* (Sheldrick, 2008 ▶); program(s) used to refine structure: *SHELXL97* (Sheldrick, 2008 ▶); molecular graphics: *PLATON* (Spek, 2003 ▶); software used to prepare material for publication: *PLATON*.

## Supplementary Material

Crystal structure: contains datablocks global, I. DOI: 10.1107/S160053680803153X/hb2811sup1.cif

Structure factors: contains datablocks I. DOI: 10.1107/S160053680803153X/hb2811Isup2.hkl

Additional supplementary materials:  crystallographic information; 3D view; checkCIF report

## Figures and Tables

**Table 1:** Hydrogen-bond geometry (Å, °)

*D*—H⋯*A*	*D*—H	H⋯*A*	*D*⋯*A*	*D*—H⋯*A*
N2—H2*C*⋯O1*W*^i^	0.86	2.06	2.906 (2)	169
N1—H1*A*⋯O1	0.86	1.82	2.676 (2)	173
N2—H2*B*⋯O2	0.86	1.98	2.826 (3)	168
O1*W*—H1*AW*⋯O2*W*	0.84	1.88	2.705 (2)	168
O1*W*—H1*BW*⋯O2	0.83	1.92	2.739 (2)	169
O2*W*—H2*AW*⋯O1*W*^ii^	0.83	1.93	2.758 (2)	172
O2*W*—H2*BW*⋯O1^iii^	0.83	1.93	2.732 (2)	160

Symmetry codes: (i) 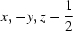; (ii) 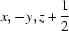; (iii) 
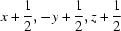
.

**Fig. 1.**
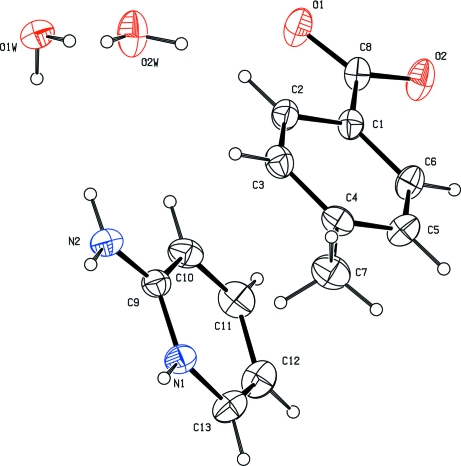
The molecular structure of (I), with displacement ellipsoids for the non-hydrogen atoms drawn at the 50% probability level.

**Fig. 2.**
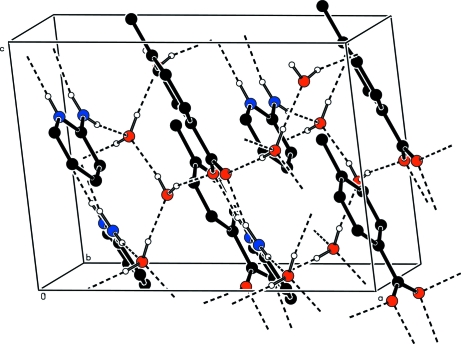
Three-dimensional structure of (I), with H bonds indicated by dashed lines. For clarity, H atoms not involved in hydrogen bonds are omitted.

## supplementary crystallographic information

### Comment

Currently, many groups are investigating supramolecular structures of cocrystals containing organic acids and organic bases resulting from hydrogen bonding (Wang & Wei, 2005). The asymmetric unit of the title compound, (I), is composed of 4-methylbenzoate anion, one 2-amino pyridinium cation and two water molecules in general positions (Fig. 1). The carboxyl group of 4-methylbenzoic acid is deprotonated. In the crystal, 2-amino pyridinium and 4-methylbenzoic acid anion together with water molecules are linked into a three-dimensional supramolecular framework by multiple N—H···O and O—H···O hydrogen bonds (Fig. 2 and Table 1).

### Experimental

4-Methylbenzoic acid (1 mmol, 0.135 g) and 2-aminopyridine (1 mmol, 0.094 g) were dissolved in 20 ml of distilled water. The solution was stirred for about 20 min at 353 K, avoiding evaporation of 2-aminopyridine. Colourless blocks of (I) were obtained from the filtrate after seven days.

### Refinement

Anomalous dispersion was negligible and Friedel pairs were merged before refinement.

The H atoms were geometrically placed with C—H = 0.93–0.96 Å, N—H = 0.86 Å and O—H = 0.83 Å, and were refined as riding with *U*_iso_(*H*)=1.2*U*_eq_(N and C_methylidyne_) and *U*_iso_(H)=1.5*U*_eq_(O or C_methyl_).

### Crystal data

**Table:**

C_5_H_7_N_2_^+^·C_8_H_7_O_2_^−^·2H_2_O	*F*(000) = 568
*M**_r_* = 266.29	*D*_x_ = 1.233 Mg m^−^^3^
Monoclinic, *C**c*	Mo *K*α radiation, λ = 0.71073 Å
Hall symbol: C -2yc	Cell parameters from 1977 reflections
*a* = 12.2059 (14) Å	θ = 2.3–26.6°
*b* = 13.1531 (16) Å	µ = 0.09 mm^−^^1^
*c* = 8.9937 (11) Å	*T* = 296 K
β = 96.617 (2)°	Block, colorless
*V* = 1434.3 (3) Å^3^	0.23 × 0.18 × 0.16 mm
*Z* = 4	

### Data collection

**Table:**

Bruker SMART CCD diffractometer	1567 independent reflections
Radiation source: fine-focus sealed tube	1406 reflections with *I* > 2σ(*I*)
graphite	*R*_int_ = 0.019
ω scans	θ_max_ = 27.0°, θ_min_ = 2.3°
Absorption correction: multi-scan (SADABS; Sheldrick, 2001)	*h* = −6→15
*T*_min_ = 0.979, *T*_max_ = 0.985	*k* = −16→16
4203 measured reflections	*l* = −11→11

### Refinement

**Table:**

Refinement on *F*^2^	Secondary atom site location: difference Fourier map
Least-squares matrix: full	Hydrogen site location: inferred from neighbouring sites
*R*[*F*^2^ > 2σ(*F*^2^)] = 0.032	H-atom parameters constrained
*wR*(*F*^2^) = 0.089	*w* = 1/[σ^2^(*F*_o_^2^) + (0.0525*P*)^2^ + 0.1161*P*] where *P* = (*F*_o_^2^ + 2*F*_c_^2^)/3
*S* = 1.04	(Δ/σ)_max_ < 0.001
1567 reflections	Δρ_max_ = 0.12 e Å^−^^3^
175 parameters	Δρ_min_ = −0.11 e Å^−^^3^
8 restraints	Extinction correction: SHELXL97 (Sheldrick, 2008), Fc^*^=kFc[1+0.001xFc^2^λ^3^/sin(2θ)]^-1/4^
Primary atom site location: structure-invariant direct methods	Extinction coefficient: 0.019 (2)

### Special details

**Table:**

Geometry. All e.s.d.'s (except the e.s.d. in the dihedral angle between two l.s. planes)are estimated using the full covariance matrix. The cell e.s.d.'s are takeninto account individually in the estimation of e.s.d.'s in distances, anglesand torsion angles; correlations between e.s.d.'s in cell parameters are onlyused when they are defined by crystal symmetry. An approximate (isotropic)treatment of cell e.s.d.'s is used for estimating e.s.d.'s involving l.s. planes.
Refinement. Refinement of *F*^2^ against ALL reflections. The weighted *R*-factor *wR* andgoodness of fit *S* are based on *F*^2^, conventional *R*-factors *R* are basedon *F*, with *F* set to zero for negative *F*^2^. The threshold expression of*F*^2^ > σ(*F*^2^) is used only for calculating *R*-factors(gt) *etc*. and isnot relevant to the choice of reflections for refinement. *R*-factors basedon *F*^2^ are statistically about twice as large as those based on *F*, and *R*-factors based on ALL data will be even larger.

### Fractional atomic coordinates and isotropic or equivalent isotropic displacement parameters (Å^2^)

**Table:**

	*x*	*y*	*z*	*U*_iso_*/*U*_eq_	
C1	0.48260 (16)	0.20609 (14)	0.6527 (2)	0.0505 (4)	
C2	0.43746 (18)	0.28865 (16)	0.7214 (2)	0.0563 (5)	
H2A	0.4359	0.3522	0.6758	0.068*	
C3	0.39515 (17)	0.27745 (17)	0.8558 (2)	0.0606 (5)	
H3A	0.3662	0.3338	0.9000	0.073*	
C4	0.39467 (17)	0.18356 (19)	0.9270 (2)	0.0607 (5)	
C5	0.4400 (2)	0.10125 (18)	0.8588 (3)	0.0682 (6)	
H5A	0.4413	0.0377	0.9044	0.082*	
C6	0.4834 (2)	0.11256 (16)	0.7238 (2)	0.0625 (6)	
H6A	0.5135	0.0565	0.6803	0.075*	
C7	0.3478 (3)	0.1719 (2)	1.0746 (4)	0.0832 (7)	
H7A	0.3541	0.1023	1.1065	0.125*	
H7B	0.2715	0.1916	1.0626	0.125*	
H7C	0.3881	0.2145	1.1485	0.125*	
C8	0.52854 (18)	0.21761 (16)	0.5061 (2)	0.0559 (5)	
C9	0.64742 (16)	0.25294 (16)	0.1096 (2)	0.0540 (5)	
C10	0.6947 (2)	0.2780 (2)	−0.0219 (3)	0.0662 (6)	
H10A	0.7279	0.2279	−0.0741	0.079*	
C11	0.6915 (2)	0.3748 (2)	−0.0715 (3)	0.0766 (7)	
H11A	0.7248	0.3912	−0.1563	0.092*	
C12	0.6383 (3)	0.4515 (2)	0.0034 (3)	0.0775 (7)	
H12A	0.6346	0.5180	−0.0321	0.093*	
C13	0.5934 (2)	0.42557 (17)	0.1272 (3)	0.0679 (6)	
H13A	0.5575	0.4747	0.1780	0.082*	
N1	0.59936 (16)	0.32882 (13)	0.17948 (19)	0.0572 (4)	
H1A	0.5713	0.3152	0.2607	0.069*	
N2	0.64687 (16)	0.16065 (14)	0.1677 (2)	0.0616 (4)	
H2B	0.6164	0.1502	0.2479	0.074*	
H2C	0.6770	0.1110	0.1252	0.074*	
O1	0.51630 (17)	0.30246 (12)	0.4395 (2)	0.0748 (5)	
O2	0.57598 (17)	0.14398 (13)	0.4549 (2)	0.0774 (5)	
O1W	0.74860 (13)	0.02177 (11)	0.56348 (16)	0.0631 (4)	
H1AW	0.7767	0.0431	0.6471	0.076*	
H1BW	0.7022	0.0649	0.5296	0.076*	
O2W	0.85359 (18)	0.06355 (15)	0.8382 (2)	0.0886 (6)	
H2AW	0.8275	0.0340	0.9081	0.133*	
H2BW	0.8906	0.1114	0.8791	0.133*	

### Atomic displacement parameters (Å^2^)

**Table:**

	*U*^11^	*U*^22^	*U*^33^	*U*^12^	*U*^13^	*U*^23^
C1	0.0524 (10)	0.0485 (10)	0.0491 (10)	0.0065 (8)	−0.0009 (8)	−0.0026 (8)
C2	0.0571 (11)	0.0491 (10)	0.0612 (12)	0.0059 (8)	0.0005 (9)	−0.0031 (9)
C3	0.0565 (11)	0.0624 (12)	0.0624 (13)	0.0081 (9)	0.0050 (10)	−0.0100 (10)
C4	0.0511 (11)	0.0738 (14)	0.0569 (13)	−0.0057 (10)	0.0045 (9)	−0.0047 (10)
C5	0.0847 (16)	0.0545 (11)	0.0661 (13)	−0.0024 (11)	0.0115 (11)	0.0063 (10)
C6	0.0797 (15)	0.0484 (10)	0.0595 (12)	0.0090 (10)	0.0086 (11)	−0.0039 (9)
C7	0.0784 (16)	0.104 (2)	0.0709 (15)	−0.0051 (15)	0.0234 (13)	0.0001 (14)
C8	0.0607 (11)	0.0550 (11)	0.0516 (10)	0.0136 (9)	0.0042 (9)	0.0013 (8)
C9	0.0496 (10)	0.0573 (11)	0.0529 (11)	0.0052 (9)	−0.0032 (9)	−0.0067 (9)
C10	0.0607 (13)	0.0826 (16)	0.0561 (12)	0.0112 (11)	0.0105 (10)	−0.0046 (11)
C11	0.0808 (17)	0.0914 (18)	0.0589 (13)	−0.0001 (13)	0.0133 (12)	0.0119 (13)
C12	0.101 (2)	0.0654 (14)	0.0632 (14)	−0.0019 (13)	−0.0014 (12)	0.0081 (11)
C13	0.0905 (16)	0.0550 (12)	0.0565 (12)	0.0086 (11)	0.0007 (11)	−0.0039 (10)
N1	0.0655 (10)	0.0568 (9)	0.0492 (9)	0.0060 (8)	0.0060 (7)	−0.0021 (7)
N2	0.0652 (10)	0.0570 (10)	0.0627 (10)	0.0110 (8)	0.0080 (8)	−0.0044 (8)
O1	0.0988 (12)	0.0615 (9)	0.0686 (10)	0.0280 (9)	0.0295 (9)	0.0155 (8)
O2	0.1095 (13)	0.0620 (9)	0.0638 (9)	0.0332 (9)	0.0228 (8)	0.0046 (7)
O1W	0.0750 (9)	0.0564 (8)	0.0593 (8)	0.0100 (7)	0.0137 (7)	−0.0006 (7)
O2W	0.1081 (15)	0.0954 (13)	0.0626 (9)	−0.0426 (12)	0.0114 (10)	−0.0026 (9)

### Geometric parameters (Å, °)

**Table:**

C1—C6	1.386 (3)	C9—N1	1.349 (3)
C1—C2	1.394 (3)	C9—C10	1.414 (3)
C1—C8	1.499 (3)	C10—C11	1.347 (4)
C2—C3	1.375 (3)	C10—H10A	0.9300
C2—H2A	0.9300	C11—C12	1.412 (4)
C3—C4	1.391 (3)	C11—H11A	0.9300
C3—H3A	0.9300	C12—C13	1.341 (4)
C4—C5	1.390 (4)	C12—H12A	0.9300
C4—C7	1.513 (4)	C13—N1	1.356 (3)
C5—C6	1.388 (4)	C13—H13A	0.9300
C5—H5A	0.9300	N1—H1A	0.8600
C6—H6A	0.9300	N2—H2B	0.8600
C7—H7A	0.9600	N2—H2C	0.8600
C7—H7B	0.9600	O1W—H1AW	0.8386
C7—H7C	0.9600	O1W—H1BW	0.8339
C8—O2	1.244 (3)	O2W—H2AW	0.8328
C8—O1	1.267 (3)	O2W—H2BW	0.8345
C9—N2	1.322 (3)		
			
C6—C1—C2	118.00 (19)	O2—C8—C1	118.96 (18)
C6—C1—C8	120.78 (17)	O1—C8—C1	117.98 (18)
C2—C1—C8	121.22 (17)	N2—C9—N1	118.26 (19)
C3—C2—C1	120.9 (2)	N2—C9—C10	124.5 (2)
C3—C2—H2A	119.5	N1—C9—C10	117.3 (2)
C1—C2—H2A	119.5	C11—C10—C9	119.9 (2)
C2—C3—C4	121.4 (2)	C11—C10—H10A	120.0
C2—C3—H3A	119.3	C9—C10—H10A	120.0
C4—C3—H3A	119.3	C10—C11—C12	121.0 (2)
C5—C4—C3	117.7 (2)	C10—C11—H11A	119.5
C5—C4—C7	121.2 (2)	C12—C11—H11A	119.5
C3—C4—C7	121.0 (2)	C13—C12—C11	117.9 (2)
C6—C5—C4	120.9 (2)	C13—C12—H12A	121.0
C6—C5—H5A	119.5	C11—C12—H12A	121.0
C4—C5—H5A	119.5	C12—C13—N1	121.1 (2)
C1—C6—C5	121.0 (2)	C12—C13—H13A	119.4
C1—C6—H6A	119.5	N1—C13—H13A	119.4
C5—C6—H6A	119.5	C9—N1—C13	122.8 (2)
C4—C7—H7A	109.5	C9—N1—H1A	118.6
C4—C7—H7B	109.5	C13—N1—H1A	118.6
H7A—C7—H7B	109.5	C9—N2—H2B	120.0
C4—C7—H7C	109.5	C9—N2—H2C	120.0
H7A—C7—H7C	109.5	H2B—N2—H2C	120.0
H7B—C7—H7C	109.5	H1AW—O1W—H1BW	106.9
O2—C8—O1	123.1 (2)	H2AW—O2W—H2BW	104.7
			
C6—C1—C2—C3	0.1 (3)	C2—C1—C8—O2	174.2 (2)
C8—C1—C2—C3	179.8 (2)	C6—C1—C8—O1	173.5 (2)
C1—C2—C3—C4	−0.7 (3)	C2—C1—C8—O1	−6.2 (3)
C2—C3—C4—C5	0.9 (3)	N2—C9—C10—C11	−179.6 (2)
C2—C3—C4—C7	−179.9 (2)	N1—C9—C10—C11	0.7 (3)
C3—C4—C5—C6	−0.5 (3)	C9—C10—C11—C12	−2.1 (4)
C7—C4—C5—C6	−179.6 (2)	C10—C11—C12—C13	1.5 (4)
C2—C1—C6—C5	0.4 (3)	C11—C12—C13—N1	0.4 (4)
C8—C1—C6—C5	−179.3 (2)	N2—C9—N1—C13	−178.5 (2)
C4—C5—C6—C1	−0.2 (4)	C10—C9—N1—C13	1.2 (3)
C6—C1—C8—O2	−6.0 (3)	C12—C13—N1—C9	−1.8 (4)

### Hydrogen-bond geometry (Å, °)

**Table:**

*D*—H···*A*	*D*—H	H···*A*	*D*···*A*	*D*—H···*A*
N2—H2C···O1W^i^	0.86	2.06	2.906 (2)	169
N1—H1A···O1	0.86	1.82	2.676 (2)	173
N2—H2B···O2	0.86	1.98	2.826 (3)	168
O1W—H1AW···O2W	0.84	1.88	2.705 (2)	168
O1W—H1BW···O2	0.83	1.92	2.739 (2)	169
O2W—H2AW···O1W^ii^	0.83	1.93	2.758 (2)	172
O2W—H2BW···O1^iii^	0.83	1.93	2.732 (2)	160

Symmetry codes: (i) *x*, −*y*, *z*−1/2; (ii) *x*, −*y*, *z*+1/2; (iii) *x*+1/2, −*y*+1/2, *z*+1/2.
